# Optimal bounds for the generalized Euler–Mascheroni constant

**DOI:** 10.1186/s13660-018-1711-1

**Published:** 2018-05-18

**Authors:** Ti-Ren Huang, Bo-Wen Han, Xiao-Yan Ma, Yu-Ming Chu

**Affiliations:** 1 Department of Mathematics, Zhejiang Sci-Tech University, Hangzhou, China; 2 Department of Mathematics, Huzhou University, Huzhou, China

**Keywords:** 11Y60, 40A05, 33B15, Euler–Mascheroni constant, gamma function, psi function, Asymptotic formula

## Abstract

We provide several sharp upper and lower bounds for the generalized Euler–Mascheroni constant. As consequences, some previous bounds for the Euler–Mascheroni constant are improved.

## Introduction

Let $a>0$. Then the generalized Euler–Mascheroni constant $\gamma (a)$ [1] is given by

$$ \gamma (a)=\lim_{n\rightarrow \infty } \biggl[ \frac{1}{a}+ \frac{1}{a+1}+ \cdots +\frac{1}{a+n-1}-\log \biggl( \frac{a+n-1}{a} \biggr) \biggr] . $$

We clearly see that the generalized Euler–Mascheroni constant $\gamma (a)$ is the natural generalization of the classical Euler–Mascheroni constant [2–5]

$$ \gamma =\gamma (1)=\lim_{n\rightarrow \infty } \biggl( 1+\frac{1}{2}+ \frac{1}{3}+\cdots +\frac{1}{n}-\log n \biggr) =0.577215664901\ldots \,. $$

Recently, the two bounds for *γ* and $\gamma (a)$ have attracted the attention of many mathematicians. In particular, many remarkable inequalities and asymptotic formulas for *γ* and $\gamma (a)$ can be found in the literature [6–10].

Let

1.1 $$\begin{aligned}& \gamma_{n}=1+\frac{1}{2}+\frac{1}{3}+\cdots + \frac{1}{n}-\log n, \\& R_{n}=1+\frac{1}{2}+\frac{1}{3}+\cdots + \frac{1}{n}-\log \biggl( n+ \frac{1}{2} \biggr) , \\& S_{n}=1+\frac{1}{2}+\frac{1}{3}+\cdots + \frac{1}{n-1}+\frac{1}{2n}- \log n, \\& T_{n}=1+\frac{1}{2}+\frac{1}{3}+\cdots + \frac{1}{n}-\log \biggl( n+ \frac{1}{2}+\frac{1}{24n} \biggr) , \\& y_{n}(a)=\frac{1}{a}+\frac{1}{a+1}+\cdots + \frac{1}{a+n-1}-\log \biggl( \frac{a+n-1}{a} \biggr) , \\& \alpha_{n}(a)=\frac{1}{a}+\frac{1}{a+1}+\cdots + \frac{1}{a+n-2}+ \frac{1}{2(a+n-1)}-\log \biggl( \frac{a+n-1}{a} \biggr) , \end{aligned}$$

1.2 $$\begin{aligned}& \beta_{n}(a)=\frac{1}{a}+\frac{1}{a+1}+\frac{1}{a+n-1}- \log \biggl( \frac{a+n-1/2}{a} \biggr) , \end{aligned}$$

1.3 $$\begin{aligned}& \lambda_{n}(a)=\frac{1}{a}+\frac{1}{a+1}+\frac{1}{a+n-1}- \log \biggl( \frac{a+n-1/2}{a}+ \frac{1}{24a(a+n-1)} \biggr) , \end{aligned}$$

1.4 $$\begin{aligned}& \mu_{n}(a)=y_{n}(a)-\frac{1}{2(a+n-1)}+\frac{1}{12(a+n-1)^{2}}- \frac{1}{120(a+n-1)^{4}}. \end{aligned}$$

Negoi [11] proved that the two-sided inequality

1.5 $$ \frac{1}{48(n+1)^{3}}\leq \gamma -T_{n}\leq \frac{1}{48n^{3}} $$

is valid for $n\geq 1$.

Qiu and Vuorinen [12] proved that the two-sided inequality

1.6 $$ \frac{1}{2n}-\frac{\lambda }{n^{2}}< \gamma_{n}-\gamma \leq \frac{1}{2n}-\frac{\mu }{n^{2}} $$

is valid for $n\geq 1$ if and only if $\lambda \geq 1/12$ and $\mu \leq \gamma -1/2$.

In [13], DeTemple proved that the double inequality

1.7 $$ \frac{1}{24(n+1)^{2}}\leq R_{n}-\gamma \leq \frac{1}{24n^{2}} $$

holds for all $n\geq 1$.

Chen [14] proved that $\alpha =1/\sqrt{12\gamma -6}-1$ and $\beta =0$ are the best possible constants such that the double inequality

1.8 $$ \frac{1}{12(n+\alpha )^{2}}\leq \gamma -S_{n}\leq \frac{1}{12(n+ \beta )^{2}} $$

holds for $n\geq 1$.

Sîntămărian [15], and Berinde and Mortici [16] proved that the double inequalities

1.9 $$\begin{aligned}& \frac{1}{2(n+a)}\leq y_{n}(a)-\gamma (a)\leq \frac{1}{2(n+a-1)}, \end{aligned}$$

1.10 $$\begin{aligned}& \frac{1}{24(n+a)^{2}}\leq \beta_{n}(a)-\gamma (a)\leq \frac{1}{24(n+a-1)^{2}} \end{aligned}$$

are valid for all $a>0$ and $n\geq 1$.

The main purpose of this article is to find the best possible constants $\alpha_{1}$, $\alpha_{2}$, $\alpha_{3}$, $\alpha_{4}$, $\beta_{1}$, $\beta_{2}$, $\beta_{3}$ and $\beta_{4}$ such that the double inequalities

$$\begin{aligned}& \frac{1}{12(a+n-\alpha_{1})^{2}}\leq \gamma (a)-\alpha_{n}(a)< \frac{1}{12(a+n- \beta_{1})^{2}}, \\& \frac{1}{24(a+n-\alpha_{2})^{2}}\leq \beta_{n}(a)-\gamma (a)< \frac{1}{24(a+n- \beta_{2})^{2}}, \\& \frac{1}{48(a+n-\alpha_{3})^{3}}\leq \gamma (a)-\lambda_{n}(a)< \frac{1}{48(a+n- \beta_{3})^{3}}, \\& \frac{\alpha_{4}}{(a+n-1)^{6}}\leq \gamma (a)-\mu_{n}(a)< \frac{\beta _{4}}{(a+n-1)^{6}} \end{aligned}$$

hold for all $a>0$ and $n\geq n_{0}$ and improve the bounds for the Euler–Mascheroni constant.

## Main results

In order to prove our main results, we need several formulas and lemmas which we present in this section.

For $x>0$, the classical gamma function Γ and its logarithmic derivative, the so-called psi function *ψ* are defined [17–24] as

$$ \Gamma (x)= \int_{0}^{\infty }t^{x-1}e^{-t}dt, \qquad \psi (x)=\frac{ \Gamma^{\prime }(x)}{\Gamma (x)}, $$

respectively.

The psi function *ψ* has the recurrence and asymptotic formulas [25] as follows:

2.1 $$\begin{aligned}& \psi (x+1)=\psi (x)+\frac{1}{x}, \end{aligned}$$

2.2 $$\begin{aligned}& \psi (x)\sim \log x-\frac{1}{2x}-\frac{1}{12x^{2}}+\frac{1}{120x^{4}}- \frac{1}{252x ^{6}}+\cdots \quad (x\rightarrow \infty ). \end{aligned}$$

### Lemma 2.1

(See [14, Proof of Theorem 1])

*The function*

2.3 $$ f_{1}(x)=\frac{1}{ \sqrt{12 ( \log x-\psi (x+1)+\frac{1}{2x} ) }}-x $$

*is strictly decreasing on* $[2, \infty )$ *with* $f_{1}(\infty )=0$.

### Lemma 2.2

(See [26, Proof of Theorem 1], [27, Remark 4])

*The function*

2.4 $$ f_{2}(x)=\frac{1}{\sqrt{24 ( \psi (x+1)-\log (x+1/2) ) }}-x $$

*is strictly decreasing on* $[2, \infty )$ *with* $f_{2}(\infty )=1/2$.

### Lemma 2.3

(See [28, Proof of Theorem 2])

*The function*

2.5 $$ f_{3}(x)=\frac{1}{\sqrt[3]{48 [ \log ( x+\frac{1}{2}+ \frac{1}{24x} ) -\psi (x+1) ] }}-x $$

*is strictly decreasing on* $[5, \infty )$ *with* $f_{3}(\infty )=83/360$.

### Lemma 2.4

(See [29, Theorem 1.2(2)])

*The function*

2.6 $$ f_{4}(x)=\frac{x^{2}}{120}- \biggl( \psi (x)-\log x+ \frac{1}{2x}+\frac{1}{12x ^{2}} \biggr) x^{6} $$

*is strictly increasing from* $(0, \infty )$ *onto* $(0, 1/252)$.

### Theorem 2.5

*Let* $\alpha_{n}(a)$ *and* $f_{1}(x)$ *be*, *respectively*, *defined by* (1.1) *and* (2.3). *Then* $\alpha_{1}=1-f_{1}(a+2)$ *and* $\beta_{1}=1$ *are the best possible constants such that the double inequality*

2.7 $$ \frac{1}{12(a+n-\alpha_{1})^{2}}\leq \gamma (a)-\alpha_{n}(a)< \frac{1}{12(a+n- \beta_{1})^{2}} $$

*holds for all* $a>0$ *and* $n\geq 3$.

### Proof

It follows from (1.1), (2.1) and (2.2) that

2.8 $$\begin{aligned} \gamma (a)-\alpha_{n}(a)&=\lim_{n\rightarrow \infty } \biggl[ \psi (n+a)- \psi (a)-\log \biggl( \frac{a+n-1}{a} \biggr) \biggr] \\ &\quad {}- \biggl[ \psi (n+a)-\psi (a)-\frac{1}{2(a+n-1)}-\log \biggl( \frac{a+n-1}{a} \biggr) \biggr] \\ &=\lim_{n\rightarrow \infty }\bigl[\psi (n+a)-\log (a+n-1)\bigr] \\ &\quad {}-\psi (n+a)+\frac{1}{2(a+n-1)}+\log (a+n-1) \\ &=\log (a+n-1)-\psi (n+a)+\frac{1}{2(a+n-1)}. \end{aligned}$$

From (2.3) and (2.8) we clearly see that inequality (2.7) is equivalent to

2.9 $$ \alpha_{1}\leq 1-f_{1}(n+a-1)< \beta_{1}. $$

Therefore, Theorem 2.5 follows easily from Lemma 2.1 and (2.19). □

### Theorem 2.6

*Let* $\beta_{n}(a)$ *and* $f_{2}(x)$ *be*, *respectively*, *defined by* (1.2) *and* (2.4). *Then* $\alpha_{2}=1-f_{2}(a+2)$ *and* $\beta_{2}=1/2$ *are the best possible constants such that the double inequality*

2.10 $$ \frac{1}{24(a+n-\alpha_{2})^{2}}\leq \beta_{n}(a)-\gamma (a)< \frac{1}{24(a+n- \beta_{2})^{2}} $$

*holds for all* $a>0$ *and* $n\geq 3$.

### Proof

It follows from (1.2), (2.1) and (2.2) that

2.11 $$ \beta_{n}(a)-\gamma (a)=\psi (n+a)-\log \biggl( a+n-\frac{1}{2} \biggr) . $$

From (2.4) and (2.11) we clearly see that inequality (2.10) can be rewritten as

2.12 $$ \alpha_{2}\leq 1-f_{2}(n+a-1)< \beta_{2}. $$

Therefore, Theorem 2.6 follows easily from Lemma 2.2 and (2.12). □

### Remark 2.1

We clearly see that both the upper and the lower bounds given in (2.10) for $\beta_{n}(a)-\gamma (a)$ are better than that given in (1.10) for $n\geq 3$ due to $1-f_{2}(2)=3-1/\sqrt{36-24( \gamma +\log 5-\log 2)}=0.466904841516\ldots $ .

### Theorem 2.7

*Let* $\lambda_{n}(a)$ *and* $f_{3}(x)$ *be*, *respectively*, *defined by* (1.3) *and* (2.5). *Then* $\alpha_{3}=1-f_{3}(a+5)$ *and* $\beta_{3}=277/360$ *are the best possible constants such that the double inequality*

2.13 $$ \frac{1}{48(a+n-\alpha_{3})^{3}}\leq \gamma (a)-\lambda_{n}(a)< \frac{1}{48(a+n- \beta_{3})^{3}} $$

*holds for all* $a>0$ *and* $n\geq 6$.

### Proof

From (1.3), (2.1) and (2.2) we have

2.14 $$ \gamma (a)-\lambda_{n}(a)=\log \biggl( a+n- \frac{1}{2}+ \frac{1}{24(a+n-1)} \biggr) -\psi (a+n). $$

It follows from (2.5) and (2.14) that inequality (2.13) can be rewritten as

2.15 $$ \alpha_{3}\leq 1-f_{3}(a+n-1)< \beta_{3}. $$

Therefore, Theorem 2.7 follows easily from Lemma 2.3 and (2.15). □

### Theorem 2.8

*Let* $\mu_{n}(a)$ *and* $f_{4}(x)$ *be*, *respectively*, *defined by* (1.4) *and* (2.6). *Then* $\alpha_{4}=f_{4}(a)$ *and* $\beta_{4}=1/252$ *are the best possible constants such that the double inequality*

2.16 $$ \frac{\alpha_{4}}{(a+n-1)^{6}}\leq \gamma (a)-\mu_{n}(a)< \frac{\beta _{4}}{(a+n-1)^{6}} $$

*holds for all* $a>0$ *and* $n\geq 1$.

### Proof

It follows from (1.4), (2.1) and (2.2) that

2.17 $$\begin{aligned} &\gamma (a)-\mu_{n}(a) \\ &\quad =\frac{1}{120(n+a-1)^{4}} \\ &\quad \quad {}- \biggl[ \psi (n+a-1)-\log (n+a-1)+ \frac{1}{2(n+a-1)}+ \frac{1}{12(n+a-1)^{2}} \biggr] . \end{aligned}$$

From (2.6) and (2.17) we clearly see that inequality (2.16) is equivalent to

2.18 $$ \alpha_{4}\leq f_{4}(n+a-1)< \beta_{4}. $$

Therefore, Theorem 2.8 follows easily from Lemma 2.4 and (2.18). □

### Remark 2.2

Note that

2.19 $$ \alpha_{n}(a)=y_{n}(a)-\frac{1}{2(a+n-1)}. $$

It follows from (1.4), Theorem 2.5, Theorem 2.8 and (2.19) that $\alpha_{1}=1-f_{1}(a+2)$, $\beta_{1}=1$, $\alpha_{4}=f_{4}(a)$ and $\beta_{4}=1/252$ are the best possible constants such that the double inequalities

2.20 $$\begin{aligned}& \frac{1}{2(a+n-1)}-\frac{1}{12(a+n-\beta_{1})^{2}}< y_{n}(a)-\gamma (a) \\& \hphantom{\frac{1}{2(a+n-1)}-\frac{1}{12(a+n-\beta_{1})^{2}}}\leq \frac{1}{2(a+n-1)}-\frac{1}{12(a+n-\alpha_{1})^{2}}, \end{aligned}$$

2.21 $$\begin{aligned}& \frac{1}{2(a+n-1)}-\frac{1}{12(a+n-1)^{2}}+\frac{1}{120(a+n-1)^{4}}-\frac{ \beta_{4}}{(a+n-1)^{6}} \\& \quad < y_{n}(a)-\gamma (a) \\& \quad \leq \frac{1}{2(a+n-1)}-\frac{1}{12(a+n-1)^{2}}+ \frac{1}{120(a+n-1)^{4}}-\frac{\alpha_{4}}{(a+n-1)^{6}}, \end{aligned}$$

hold for all $a>0$ and $n\geq 3$.

We clearly see that the two inequalities (2.20) and (2.21) are the improvements of the inequality (1.9) for $n\geq 3$.

Let $a=1$ and

$$\begin{aligned}& c_{1}=f_{1}(3)=1/\sqrt{12(\gamma +\log 3)-20}-3=0.015998 \ldots \,, \\& c_{2}=f_{2}(3)=1/ \sqrt{44-24(\gamma +\log 7-\log 2)}-3=0.5242567\ldots \,, \\& c_{3}=f_{3}(6)=-6+1/\sqrt[3]{48(\gamma -49/20+\log 937-\log 144)}=0.242347\ldots \end{aligned}$$

and

$$\begin{aligned}& c_{4}=f_{4}(1)=\gamma -23/40=0.00221566\ldots \,. \end{aligned}$$

Then

$$\begin{aligned}& \gamma (1)=\gamma , \qquad \alpha_{n}(1)=\gamma_{n}- \frac{1}{2n}=S_{n}, \qquad \beta_{n}(1)=R_{n}, \\& \lambda_{n}(1)=T_{n}, \qquad \mu_{n}(1)= \gamma_{n}-\frac{1}{2n}+\frac{1}{12n ^{2}}-\frac{1}{120n^{4}}. \end{aligned}$$

Therefore, Theorems 2.5–2.8 lead to Corollaries 2.1–2.5 immediately.

### Corollary 2.1

*The double inequality*

2.22 $$ \frac{1}{2n}-\frac{1}{12n^{2}}< \gamma_{n}-\gamma \leq \frac{1}{2n}-\frac{1}{12(n+c _{1})^{2}} $$

*holds for all* $n\geq 3$.

### Corollary 2.2

*The double inequality*

2.23 $$ \frac{1}{12(n+c_{1})^{2}}\leq \gamma -S_{n}< \frac{1}{12n^{2}} $$

*holds for all* $n\geq 3$.

### Corollary 2.3

*The double inequality*

2.24 $$ \frac{1}{24(n+c_{2})^{2}}\leq R_{n}-\gamma < \frac{1}{24(n+1/2)^{2}} $$

*holds for all* $n\geq 3$.

### Corollary 2.4

*The double inequality*

2.25 $$ \frac{1}{48(n+c_{3})^{2}}\leq \gamma -T_{n}< \frac{1}{48(n+83/360)^{2}} $$

*holds for all* $n\geq 6$.

### Corollary 2.5

*The double inequality*

2.26 $$ \frac{1}{2n}-\frac{1}{12n^{2}}+\frac{1}{120n^{4}}-\frac{1}{252n^{6}} < \gamma_{n}-\gamma \leq \frac{1}{2n}-\frac{1}{12n^{2}}+ \frac{1}{120n ^{4}}-\frac{c_{4}}{n^{6}} $$

*holds for all* $n\geq 1$.

### Remark 2.3

We clearly see that the upper bound given in (2.22) is better than that given in (1.6) for $n\geq 3$ due to $n>\sqrt{12(\gamma -1/2)}c_{1}/(1-\sqrt{12(\gamma -1/2)})=0.4117\ldots $ is the solution of the inequality $1/[12(n+c_{1})^{2}]>( \gamma -1/2)/n^{2}$, the lower bound given in (2.23) is better than that given in (1.8) for $n\geq 3$ due to $c_{1}<1\sqrt{12\gamma -6}-1=0.03885914\ldots$ , both the upper and the lower bounds given in (2.24) are improvements of that given in (1.7) for $n\geq 3$, inequality (2.25) is stronger than inequality (1.5) for $n\geq 6$, the lower bound given in (2.26) is better than that given in (1.6) for $n\geq 1$, and the upper bound given in (2.26) is stronger than that given in (1.6) for $n\geq 2$ due to

$$ n> \biggl( \frac{1+\sqrt{1-4800[1-12(\gamma -1/2)]c_{4}}}{20[1-12( \gamma -1/2)]} \biggr) ^{1/2}=1.00000000006823\ldots $$

being the solution of the inequality

$$ \frac{1}{2n}-\frac{1}{12n^{2}}+\frac{1}{120n^{4}}-\frac{c_{4}}{n^{6}}< \frac{1}{2n}-\frac{\gamma -1/2}{n^{2}}. $$

## Results and discussion

As the natural generalization of the Euler–Mascheroni constant

$$ \gamma =\lim_{n\rightarrow \infty } \biggl( 1+\frac{1}{2}+ \frac{1}{3}+ \cdots +\frac{1}{n}-\log n \biggr) =0.5772156649\ldots\, , $$

the generalized Euler–Mascheroni constant is defined by

$$ \gamma (a)=\lim_{n\rightarrow \infty } \biggl[ \frac{1}{a}+ \frac{1}{a+1}+ \cdots +\frac{1}{a+n-1}-\log \biggl( \frac{a+n-1}{a} \biggr) \biggr] $$

for $a>0$.

Recently, the evaluations for *γ* and $\gamma (a)$ have been the subject of intensive research. In the article, we provide several sharp upper and lower bounds for the generalized Euler–Mascheroni constant $\gamma (a)$. As applications, we improve some previously results on the Euler–Mascheroni constant *γ*. The idea presented may stimulate further research in the theory of special function.

## Conclusion

In this paper, we present several best possible approximations for the generalized Euler–Mascheroni constant

$$ \gamma (a)=\lim_{n\rightarrow \infty } \biggl[ \frac{1}{a}+ \frac{1}{a+1}+ \cdots +\frac{1}{a+n-1}-\log \biggl( \frac{a+n-1}{a} \biggr) \biggr] $$

and improve some well-known bounds for the Euler–Mascheroni constant,

$$ \gamma =\lim_{n\rightarrow \infty } \biggl( 1+\frac{1}{2}+ \frac{1}{3}+ \cdots +\frac{1}{n}-\log n \biggr) =0.5772156649\ldots\,. $$
